# The landscape and plasticity of transposable element expression in an adult stem cell of high physiological and clinical significance

**DOI:** 10.1038/s41420-026-03277-7

**Published:** 2026-08-04

**Authors:** Xingyu Fu, Qiangwei Cai, Jingjing Zhao, Ying Chen, Jiangping He, Ke Hu, Yi Yin, Wenhan Yang, Lian Wang, Jiayi Zhao, Shuo Wu, Fei He, Zhenzhou Li, Jiekai Chen, Keqiang Zuo, Xianghuai Xu, Huan Wang, Peibin Dai, Zhengliang Gao

**Affiliations:** 1 Shanghai Baoshan Luodian Hospital, School of Medicine, Shanghai University, Shanghai, 201908 China; 2 Department of Anesthesiology and Perioperative Medicine, General Hospital of Ningxia Medical University, Ningxia, 750004 China; 3 Department of Cardiac and Vascular Surgery, Huaihe Hospital of Henan University, 115 Ximen Street, Kaifeng, Henan 475000 China; 4 Department of Anesthesiology, Gongli Hospital of Shanghai Pudong New Area, Shanghai, 200135 China; 5 Department of General Practice, Shanghai 411 Hospital, China RongTong Medical Healthcare Group Co., Ltd., Shanghai, 200081 China; 6 Guangzhou National Laboratory, Guangzhou, 510530 China; 7 Department of Pulmonary and Critical Care Medicine, Tongji Hospital, School of Medicine, Tongji University, Shanghai, 200065 China; 8 Center for Biomedical Digital Science, Guangdong Provincial Key Laboratory of Stem Cell and Regenerative Medicine, Guangdong-Hong Kong Joint Laboratory for Stem Cell and Regenerative Medicine, Guangzhou Institutes of Biomedicine and Health, Chinese Academy of Sciences, Guangzhou, 510530 China; 9 Department of Pulmonary and Critical Care Medicine, Shanghai Pulmonary Hospital, School of Medicine, Tongji University, Shanghai, China; 10 China-Japan Friendship Medical Research Institute, School of Medicine, Shanghai University, Shanghai, 200444 China

**Keywords:** Mesenchymal stem cells, Mesenchymal stem cells

## Abstract

Transposable elements (TEs) contribute to diverse aspects of stem cell biology, particularly in germline and pluripotent stem cells, which typically exhibit more open chromatin. In human adult stem cells (ASCs), however, a comprehensive framework describing the landscape and plasticity of TE expression and how these patterns may inform studies of ASC biological properties and considerations relevant to clinical translations has been lacking. Here, using human adipose-derived mesenchymal stem cells (hADSCs) as an adult stem cell paradigm, we combined transcriptomic profiling with integrative bioinformatics to map the TE expression landscape in hADSCs across multiple biologically and manufacturing-relevant contexts. We examined TE expression dynamics across culture conditions, senescence, and donor-related backgrounds, and found that TE expression patterns in hADSCs were highly plastic in response to environmental cues. Comparative analyses of TE and gene expression showed that TE patterns and dynamics correlated with transcriptomic signatures related to mesenchymal stem/stromal cells (MSCs) biological states. HERV16-int, L1MEh, and MER61E emerged as recurrent, context-responsive TE candidates associated with transcriptional signatures related to MSC immune-related, stemness-related, and senescence-related programs. This work addresses a gap in MSC TE expression profiling and provides a framework for prioritizing candidate TE-expression features with potential relevance to future MSC manufacturing and quality-assessment studies, pending orthogonal and functional validation.

## Introduction

Transposable elements (TEs) are mobile genetic elements that constitute approximately 50% of the human genome. They are increasingly recognized for their complexity and for their roles in gene regulation and genome evolution [1]. Because TE activity can be both functional and potentially deleterious, TE expression and mobilization are tightly regulated [2–6]. When dysregulated, TEs have been linked to cancer, aging, and autoimmune diseases [7], and may serve as disease biomarkers [8, 9]. In cancer, TEs drive oncogenesis through hypomethylation-induced chimeric transcripts, alternative promoters, and tumor-specific antigens [10–15]; in aging, retrotransposon reactivation has been associated with inflammatory programs and senescence-related feedback [16–19]. In viral or inflammatory conditions (e.g., COVID-19 and rheumatoid arthritis), aberrant TE activity has also been reported to promote immune activation [7, 20].

Emerging evidence further suggests that TEs participate in biological processes relevant to stem cells and development [1, 21]. TEs are broadly expressed in pluripotent stem cells, including embryonic stem cells (ESCs) and induced pluripotent stem cells (iPSCs), where they can influence pluripotency, differentiation, and developmental programs [4, 22–30]. In contrast, for most adult tissues, systematic studies remain limited regarding how TE expression is developmentally regulated and how TE dynamics relate to adult stem cell properties and cellular states. It is particularly troubling that, as we enter the era of cell therapy, although several studies have assessed retrotransposition, regulation, and activation of TEs in various adult stem cells [31], investigations into how the dynamic expression landscapes of different TE classes during stem cell manufacturing and clinical application may be associated with stem cell properties and safety-relevant considerations remain limited.

Mesenchymal stem/stromal cells (MSCs) are fibroblast-like cells present in nearly all tissues [32–34]. MSCs secrete diverse extracellular matrix components and factors to support neighboring parenchymal cells and are crucial in tissue development, homeostasis, aging, and diseases [35]. For their versatile tissue-supporting and microenvironment-modulating roles, MSCs have become leading candidates for regenerative medicine, with numerous clinical trials conducted to date but variable efficacy overall [36, 37]. MSCs can be isolated from diverse tissue sources and exhibit substantial heterogeneity influenced by donor genetic background, age, health status, anatomical origin, and isolation and culture methods [32, 33, 38]. While extensive transcriptomic efforts have examined MSC commonality, heterogeneity, and plasticity, the potential contributions and complications associated with TE expression remain underexplored.

Using hADSCs as an MSC paradigm, we aimed to characterize the landscape and plasticity of TE expression in human adult stem cells. We focused on manufacturing-relevant variables, including culture medium composition, growth factor exposure, donor context, and senescence-associated conditions. By systematically analyzing TE expression dynamics across these settings, we sought to establish an associative and hypothesis-generating framework for defining TE-associated transcriptional states in hADSCs. This framework helps define recurrent and context-responsive TE-expression features, providing a systematic basis for prioritizing candidate TEs for future mechanistic and functional investigations. It may also support the identification of candidate transcriptomic indicators for MSC process development, quality assessment, and potential safety evaluations related to MSC culture states and bioprocessing contexts.

## Results

### Global expression features of TEs in hADSCs

**Fig. 1:**
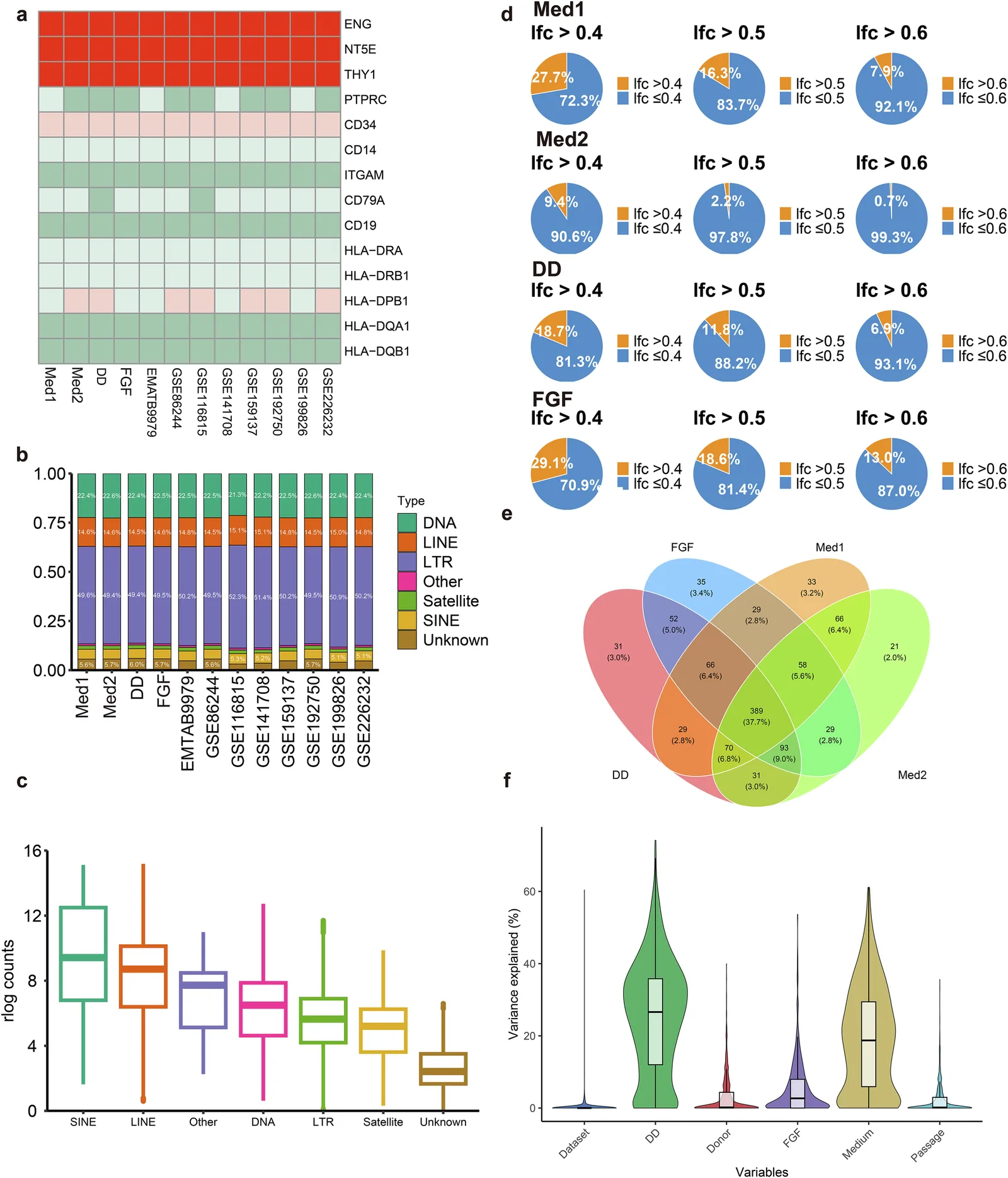
TE expression in hADSCs is globally stable across datasets but shaped by experimental context. **a** Heatmap of canonical MSC marker expression across in-house (Med1, Med2, DD, and FGF) and public hADSC datasets, confirming hADSC identity. **b** Stacked bar chart showing the proportions of detected TE types in each dataset. **c** Box plots showing regularized log-transformed expression of different TE classes across all samples. **d** Pie charts showing the proportions of TEs with limited or higher variability across Med1, Med2, DD, and FGF, based on max–min log2 fold change thresholds of >0.4, >0.5, or >0.6. **e** Venn diagram showing the overlap of low-variability TEs across the four datasets. **f** Variance decomposition of TE expression in the integrated in-house cohort (*n* = 95), showing the fractions of variance explained by dataset, DNA damage, donor, FGF2, medium, and passage. TE expression variability is shaped by experimental context, particularly DNA damage, culture medium, and FGF2 exposure.

We performed four experiments (Supplementary Fig. 1a) and generated four datasets: Medium Experiment 1 (Med1, *n* = 20), Medium Experiment 2 (Med2, *n* = 16), DNA Damage Experiment (DD, *n* = 24), and FGF2 Experiment (FGF, *n* = 35). We also analyzed eight public hADSC datasets (E-MTAB-9979, GSE86244, GSE116815, GSE141708, GSE159137, GSE192750, GSE199826, GSE226232). Samples with an overall alignment rate exceeding 85% were selected for analysis (Supplementary Fig. 1b). Robust expression of CD105/ENG, CD73/NT5E, and CD90/THY1 together with low expression of CD34, CD14, CD19, CD45/PTPRC, and HLA, supported hADSC identity in all samples (Fig. 1a, Supplementary Fig. 1d). The numbers and types of expressed TEs remained consistent across datasets (Fig. 1b), and the information on TEs is provided in Supplementary Table 1. SINE and LINE elements were generally more highly expressed, whereas LTR elements showed lower expression levels (Fig. 1c), consistent with known TE expression characteristics.

Under the family-level TE count strategy, TE expression showed smaller global shifts than protein-coding genes. To contextualize meaningful TE changes, we computed the log_2_ fold change (LFC) between the maximum and minimum expression levels for each TE across the four experimental datasets. Approximately 70% of TEs had a LFC below 0.4 (Fig. 1d), providing a reference point for defining constant versus differential expression of TEs. We then calculated residuals for each TE in each dataset and identified elements with minimal expression variability after rlog-based mean–variance trend correction (Supplementary Table 2). Cross-comparison of low-residual TEs (defined as the top 70% with the smallest residual variance in each dataset) identified 389 TEs with consistently low variability across all four experimental settings (Fig. 1e; Supplementary Table 2). Endogenous retroviruses (ERVs) constituted the largest fraction of these low-variability TEs (Supplementary Fig. 1e), consistent with reports that most ERVs remain transcriptionally repressed in human cells [39]. We next performed variance decomposition on the four integrated datasets as validated by consistent housekeeping gene (HKG) expression (Supplementary Fig. 1c). Sample sizes for each factor are listed in Supplementary Table 3. Induced DNA damage accounted for the largest fraction of variance, followed by culture medium, FGF2, donor identity, and passage within the analyzed cohort (Fig. 1f).

### Medium-dependent TE expression plasticity in hADSCs

**Fig. 2:**
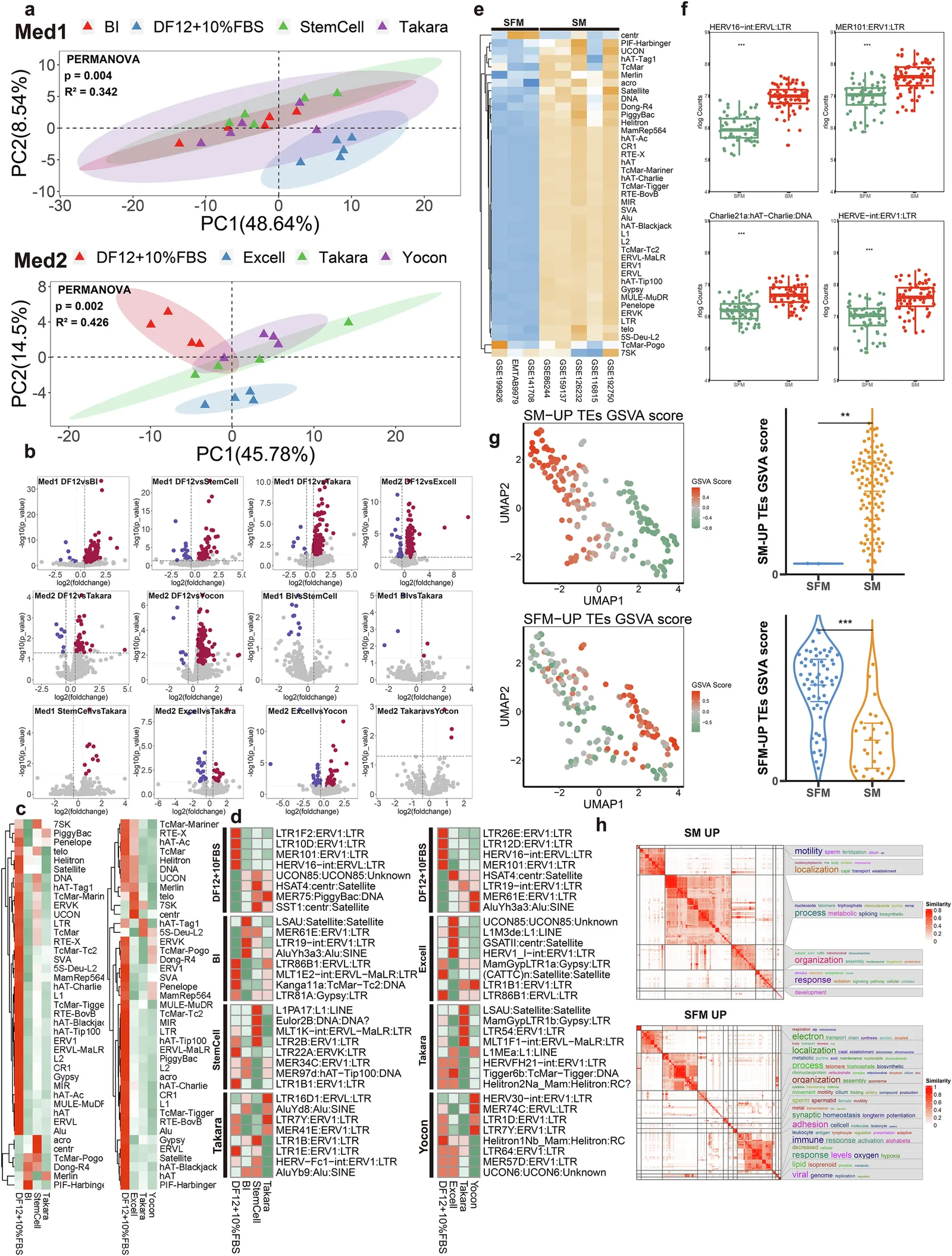
Serum-containing media are associated with higher overall TE expression in hADSCs. **a** PCA of TE expression in Med1 (BI, DF12, StemCell, and Takara) and Med2 (DF12, Excell, Takara, and Yocon), showing separation by culture medium. *P* values and R^2^ from PERMANOVA are indicated. **b** Volcano plots of pairwise differential TE expression between the indicated media in Med1 and Med2. Significantly altered TEs were defined as baseMean > 10, adjusted *P* < 0.05, and |log2FC | > 0.4. **c** Heatmaps showing group-averaged TE family expression across media in Med1 (left) and Med2 (right). SM-cultured hADSCs showed higher overall TE expression. **d** Heatmaps of TEs distinguishing serum-containing media (SM) and serum-free media (SFM) in Med1 (left) and Med2 (right). **e** Heatmap showing TE family expression across public hADSC datasets cultured under SM and SFM conditions. **f** Box plots showing expression of representative SM-associated TEs (HERV16-int, MER101, Charlie21a, and HERVE-int) in the integrated SM and SFM groups. Significance was assessed by two-sided Wilcoxon rank-sum test; ***P.adj ≤ 0.001, **P.adj ≤ 0.01, *P.adj ≤ 0.05, and NS, not significant. **g** GSVA scores of SM-upregulated and SFM-upregulated TE sets shown on UMAP embeddings and as violin plots across all samples. **h** Semantic similarity clustering of enriched GO Biological Process terms derived from genes associated with DETEs between SM and SFM conditions. Heatmaps in **c** and **d** are shown as row-scaled, with red and green indicating relatively high and low expression, respectively; in **e**, yellow and blue indicate relatively high and low expression, respectively.

MSCs exhibit distinct gene expression profiles when cultured in different media in vitro [40, 41]. We asked whether TE expression showed similar medium-dependent plasticity. Principal component analysis (PCA) of Med1 and Med2 showed distinct gene expression profiles in each medium (Supplementary Fig. 2a), with TE expression displaying similar but weaker media-specific differences (*P* < 0.05; Fig. 2a).

Differential expression analysis in Med1 and Med2 indicated that hADSCs cultured in fetal bovine serum-containing medium (SM) exhibited more differentially ex`pressed TEs (DETEs) than other conditions, with most DETEs upregulated (Fig. 2b). In contrast, serum-free media (SFM) conditions yielded fewer DETEs. The total expression of each TE superfamily also showed that samples cultured in SM exhibited globally higher TE expression, whereas other media were associated with more modest increases (Fig. 2c).

hADSCs cultured in different media exhibited distinct TE profiles in both experiments. Specific TEs (e.g., MER101, HERV16-int) were upregulated in SM (Fig. 2d). Public datasets supported this pattern: SM- and SFM-cultured samples tended to cluster separately (Supplementary Fig. 2b), and SM samples showed globally higher TE expression (Fig. 2e).

Differential expression analysis across multiple SM vs. SFM comparisons identified four consistently upregulated TEs in SM (HERV16-int, MER101, Charlie21a, and HERVE-int; Fig. 2f, Supplementary Fig. 2c). Recognizing that the TE counting strategy might cause some TEs to escape statistical significance, we applied a lenient threshold ( | log_2_FC | > 0.4 and baseMean > 10), which yielded 53 shared DETEs, 51 of which were SM-upregulated (Supplementary Fig. 2d). Gene Set Variation Analysis (GSVA) scores for condition-specific TE sets were significantly higher in their corresponding sample groups (Fig. 2g; Supplementary Fig. 2e).

We next applied an association-based enrichment framework (TEBFE; Methods) to summarize biological processes recurrently linked to genes correlated with the DETEs. GO biological process (GO BP) semantic enrichment among genes associated with the 53 shared DETEs suggested that SFM-upregulated TEs correlated with genes involved in telomere-related processes, metabolism, and Cajal body-associated terms. In contrast, SM-upregulated TEs correlated with immune, neural, and spermatogenesis-related terms (Fig. 2h). Ranking enriched GO BP terms by adjusted *p* values based on term frequency yielded consistent results. The most recurrently associated genes are shown in Supplementary Fig. 2f,g.

### TE expression patterns associated with FGF2 exposure in hADSCs

**Fig. 3:**
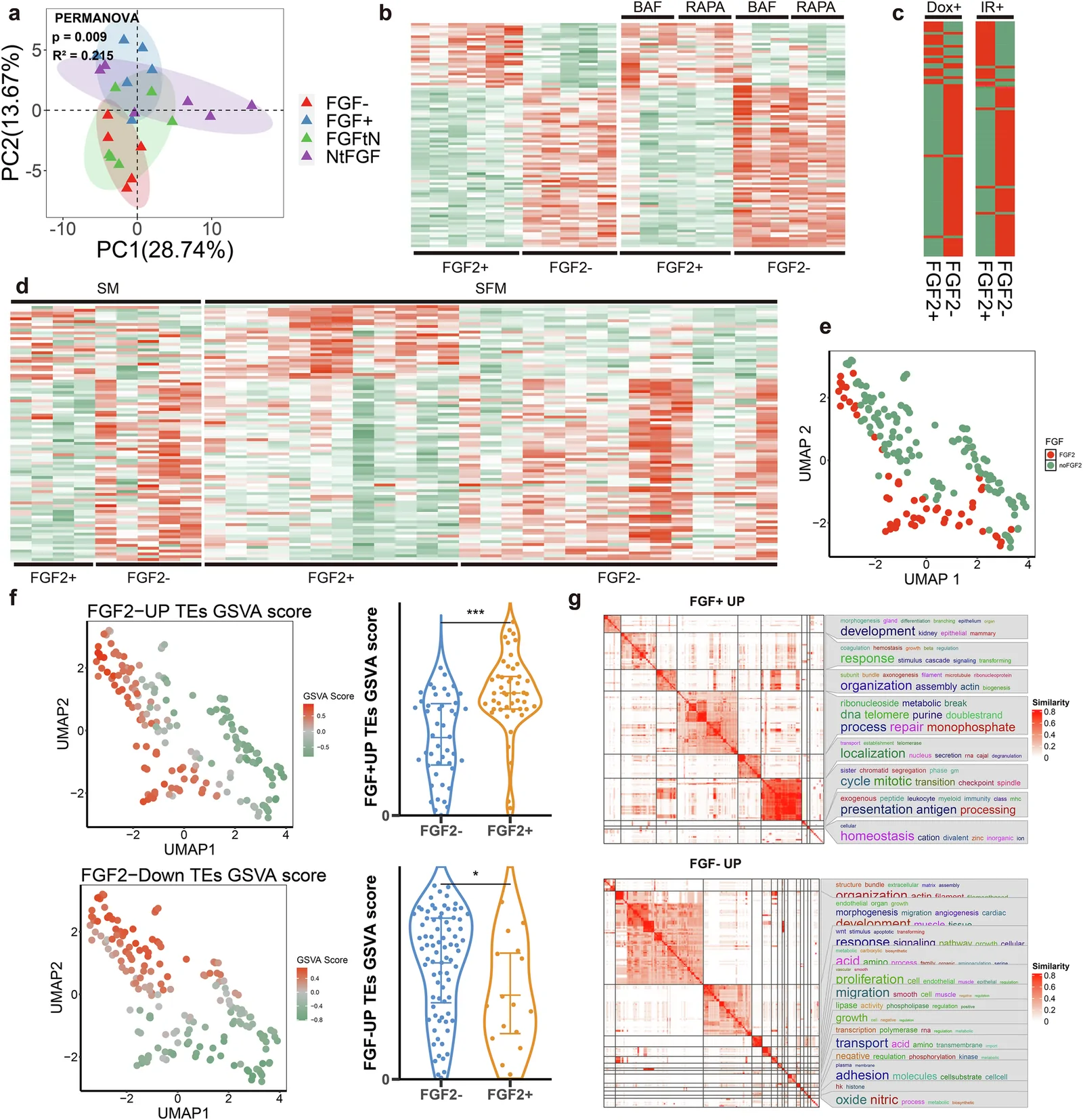
FGF2 exposure is associated with recurrent TE-expression patterns in hADSCs. **a** PCA of TE expression in the FGF dataset, showing separation by FGF2-related culture condition (FGF − , FGF + , FGFtN, and NtFGF). *P* values and R² from PERMANOVA are indicated. **b** Heatmaps showing the 90 differentially expressed TEs (DETEs) identified in the FGF dataset. Left, samples in the FGF dataset grouped by FGF2 status; right, BAF- and RAPA-treated samples further grouped by FGF2 status. FGF2-associated TE patterns are reproducible across many contexts. **c** Heatmap showing the same 90 DETEs in the DD dataset under Dox+ and IR+ conditions, grouped by FGF2 status. **d** Heatmap showing the same 90 DETEs in the Med1 and Med2 datasets, grouped by serum condition (SM or SFM) and FGF2 status. In **b**–**d**, rows represent DETEs and columns represent samples or groups; red, white, and green indicate relatively high, intermediate, and low expression, respectively. **e** UMAP of all samples colored by FGF2 status. **f** GSVA scores of FGF2-up and FGF2-down TE sets shown on UMAP projections and as violin plots across samples. **g** Semantic enrichment of GO Biological Process terms associated with DETEs between FGF2+ and FGF2− conditions. Upper and lower panels show semantic similarity matrices for genes associated with TEs upregulated in FGF2+ and FGF2− conditions, respectively; accompanying word clouds summarize representative enriched processes.

Fibroblast growth factor 2 (FGF2) is one of the most commonly used additives during MSC culture. Hence, we utilized FGF2 as a model to examine whether secreted factor treatments reshape TE expression in hADSCs. PCA revealed separation of TE expression profiles by FGF2 status (*P* < 0.05 and R^2^ = 0.215; Fig. 3a). Differential expression analysis identified 90 DETEs (26 upregulated and 64 downregulated in the FGF2+ condition; Fig. 3b). The FGF2 addition and removal assays supported a contribution of FGF2 exposure to these TE-expression changes: addition suppressed the downregulated TE set and enhanced the upregulated set, whereas withdrawal reversed these patterns (Supplementary Fig. 3a).

We next asked whether FGF2-associated TE-expression patterns remained detectable across additional perturbation settings. When hADSCs were exposed to cellular stress via autophagy-related agents (rapamycin, RAPA; bafilomycin A1, BAF), the FGF2-associated TE patterns were largely preserved (Fig. 3b). In the DD dataset, TE expression patterns of these DETEs were also observed under Dox+ and IR+ conditions (Dox+ and IR + ; Fig. 3c). Similar patterns were observed across Med1 and Med2 (Fig. 3d). GSVA scores based on DETE sets showed consistent trends across samples (Fig. 3e, f).

The TEBFE analysis of these TEs showed the top semantic enrichment keywords were telomere maintenance, DNA repair, and cell cycle. The most frequently enriched GO BP terms of genes correlated with DETEs in FGF2+ cultured hADSCs were related to DNA replication and ribosome biogenesis (Fig. 3g; Supplementary Fig. 3b); the most recurrently associated genes included HMGA2 and ADGRG1, aligning with transcriptional programs related to FGF2-supported MSC proliferation. In parallel, the top enriched keywords for genes associated with TEs downregulated by FGF2 were related to tissue development, morphogenesis, and cell metabolism, and processes related to extracellular matrix organization and muscle cell differentiation were frequently enriched (Fig. 3g; Supplementary Fig. 3c).

### Donor heterogeneity is associated with TE expression in hADSCs cultured in vitro

**Fig. 4:**
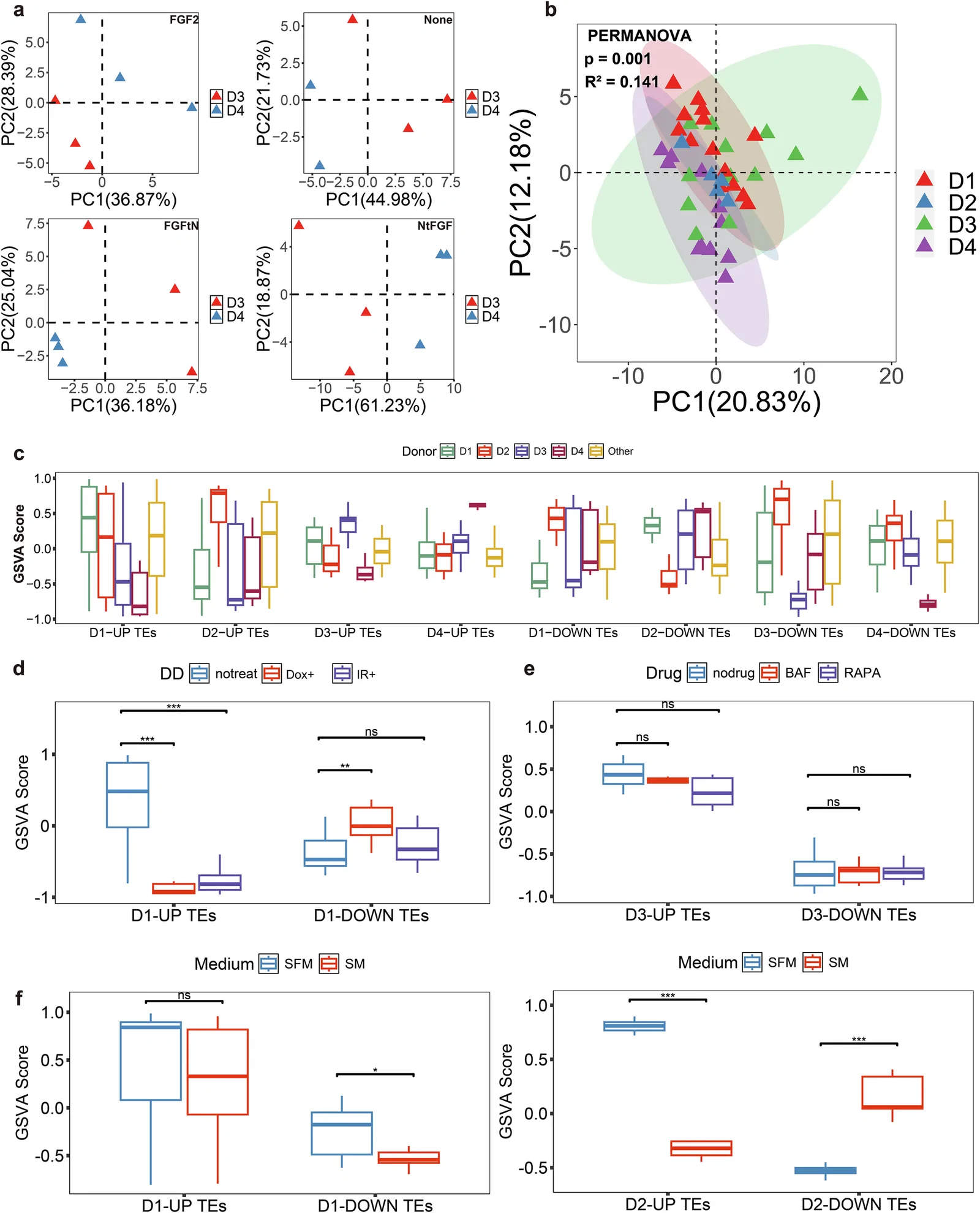
Donor-associated TE expression patterns are modulated by in vitro culture conditions. **a** PCA of TE expression in the FGF dataset under the indicated FGF2 culture conditions, colored by donor identity, showing donor-related clustering. **b** PCA of TE expression across hADSC samples cultured in serum-containing medium without strong perturbation, colored by donor identity. Shaded ellipses indicate donor-wise sample dispersion; *P* values and *R*² from PERMANOVA are indicated. **c** Box plots showing GSVA scores of donor-associated TE sets across donors. D1-UP/DOWN, D2-UP/DOWN, D3-UP/DOWN, and D4-UP/DOWN denote TE sets upregulated or downregulated in the indicated donor relative to the other donors. **d**–**f** Box plots showing GSVA scores of donor-associated TE sets across different experimental contexts: **d** D1-UP and D1-DOWN TE sets in the DNA-damage dataset under untreated, Dox + , and IR+ conditions; **e** D3-UP and D3-DOWN TE sets in the BAF/RAPA dataset under untreated, BAF-treated, and RAPA-treated conditions; and **f** D1-UP/D1-DOWN together with D2-UP/D2-DOWN TE sets under serum-free medium (SFM) and serum-containing medium (SM) conditions. Higher GSVA scores indicate stronger enrichment of the indicated donor-associated TE set. In all box plots, center lines indicate medians, boxes indicate interquartile ranges, and statistical significance is shown in the plots. Donor-associated TE signatures can be attenuated or reshaped by culture and perturbation contexts.

Donor-associated variability is a critical and understudied source of heterogeneity in ASC culture and manufacturing contexts. In the FGF dataset, TE expression profiles of samples from the same donor tended to form relatively tighter clusters (D3, D4; Supplementary Fig. 3a; Fig. 4a), motivating a more detailed assessment.

All samples in our experiments derived from four donors (Supplementary Table 3). To minimize the influence of culture medium and strong perturbation, we selected only hADSC samples cultured in SM and without intense treatment. PCA revealed donor-associated variation in TE expression within the analyzed cohort (Fig. 4b). Differential expression analysis further indicated detectable donor-associated variation in TE expression (Supplementary Table 4; Supplementary Fig. 4a).

Donor-associated DETEs exhibited class-level differences: donor D3 showed a higher proportion of LINE elements, whereas donor D1 showed more satellite elements (Supplementary Fig. 4b). PCA indicated that including these TEs in the analysis improved donor separation, while excluding them from the analysis reduced inter-donor variability (Supplementary Fig. 4c, d). Nonetheless, both stable and variable TE sets were associated with GO enrichments related to mitochondrial function, ribosomal processes, and RNA processing (Supplementary Fig. 4e,f), suggesting potential donor-related metabolic or cellular-state differences.

### Donor-associated TE expression patterns in hADSCs are modifiable under in vitro culture conditions

To quantify donor-associated TE expression patterns, we computed GSVA scoring in all samples using the donor-associated DETE sets (Fig. 4c). The donor-associated DETE sets distinguished donor samples within the baseline culture context. However, donor specificity was attenuated in a subset of D1 samples, which corresponded to 2-day Dox or IR treatments (Fig. 4d): D1-upregulated TE sets showed reduced GSVA scores, whereas D1-downregulated TE sets increased.

Given the well-documented sensitivity of hADSC transcriptional programs to culture conditions, we tested whether environmental changes could alter donor-associated TE expression in hADSCs (donor distribution across conditions is provided in Supplementary Table 3). Both BAF and RAPA treatments attenuated donor-associated TE expression patterns (Fig. 4e). Reanalysis of donor-associated DETEs confirmed that while these TEs could distinguish donor samples within this cohort, their expression patterns were modifiable under culture conditions (Supplementary Fig. 4a), consistent with GSVA scoring results (Fig. 4f).

### hADSC senescence is accompanied by changes in TE expression

Senescence during in vitro expansion is a major bottleneck for ASC applications, and TE dysregulation has been widely associated with senescent states. In this study, we utilized three senescence models: doxorubicin (Dox)-induced senescence, irradiation (IR)-induced senescence, and replicative senescence induced by passaging.

**Fig. 5:**
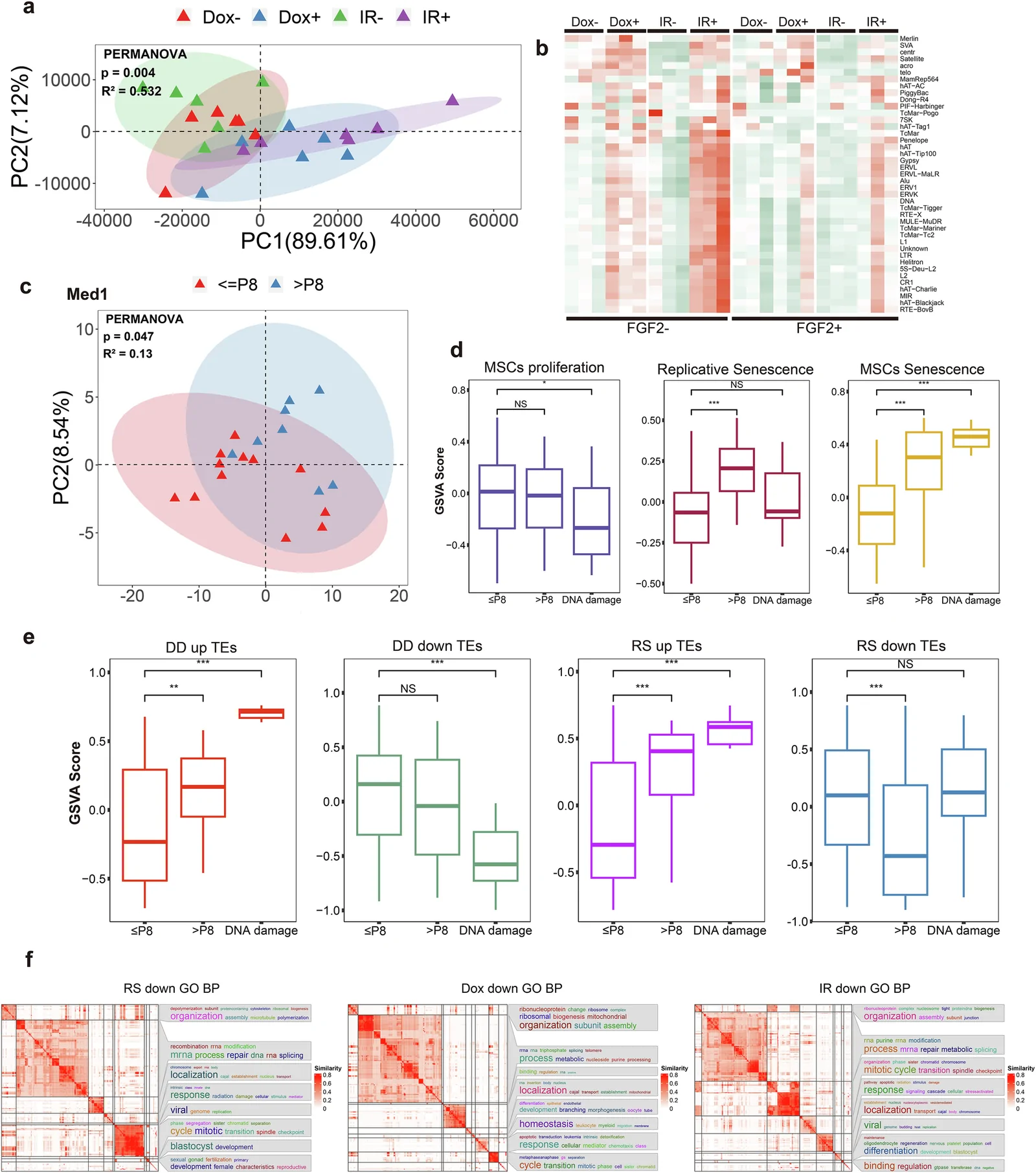
Senescence/aging-associated TE expression changes. **a** PCA of TE expression in the DNA-damage dataset, showing separation of samples by Dox and IR treatment. *P* values and *R*² from PERMANOVA are indicated. **b** Heatmap showing global TE family expression in hADSCs before and after Dox or IR treatment, stratified by FGF2 status. **c** PCA of TE expression in the Med1 dataset grouped by passage number ( ≤ P8 and >P8). *P* values and *R*² from PERMANOVA are indicated. **d** Box plots showing GSVA scores of gene sets related to MSC proliferation, replicative senescence, and MSC senescence across the ≤P8, >P8, and DNA-damage groups. **e** Box plots showing GSVA scores of DNA-damage-associated (DD up and DD down) and replicative senescence-associated (RS up and RS down) TE sets across the ≤P8, >P8, and DNA-damage groups. Higher GSVA scores indicate stronger enrichment of the indicated gene set or TE set. **f** Semantic enrichment of GO Biological Process terms associated with DETEs downregulated in RS, Dox + , and IR+ groups. Heatmap colors indicate semantic similarity, and the accompanying word clouds summarize representative enriched processes. In **b**, red, white, and green indicate relatively high, intermediate, and low expression, respectively. In **d** and **e**, center lines indicate medians, boxes indicate interquartile ranges, and statistical significance is shown in the plots (***P ≤ 0.001, **P ≤ 0.01, *P ≤ 0.05, NS, not significant).

Like genes, TE expression globally underwent a dramatic shift upon Dox and IR treatment (Fig. 5a; Supplementary Fig. 5a), with a smaller magnitude of change in hADSCs cultured with FGF2 than in those without FGF2 (Fig. 5b). Differential expression analysis identified 115 upregulated and 21 downregulated TEs for the Dox+ group and 659 upregulated and 15 downregulated TEs (Supplementary Table 5) for the IR+ group. Similarly, both gene and TE expression underwent a progressive change with replicative senescence as hADSC samples from early (P4-8) and late passages (P10, P12) clustered into distinct groups (*P* < 0.05; Fig. 5c; Supplementary Fig. 5b). Late-passage cells showed 50 upregulated and 3 downregulated TEs (Supplementary Fig. 5c; Supplementary Table 5). Because senescence induced broad transcriptome remodeling, these TE changes may reflect stress-driven transcriptional shifts rather than TE-specific responses. Fold-change distributions showed that Dox/IR shifted coding genes bidirectionally, whereas TE changes were predominantly increased (Supplementary Fig. 5d).

GSVA analysis of senescence-associated gene and TE sets revealed distinct dynamics. An MSC proliferation gene set decreased, whereas a senescence gene set increased in late-passage and DNA-damaged samples (Fig. 5d). A replicative senescence gene set increased specifically in late passages, suggesting divergent pathways between replicative and damage-induced senescence. For TE sets, upregulated DETEs increased progressively with both passage and DNA damage, while downregulated DETEs decreased (Fig. 5e). Replicative senescence-associated upregulated TEs were also increased in damage models, but corresponding downregulated TEs showed replicative senescence model-specific patterns.

TEBFE analysis suggested that in control groups ( ≤ P8, Dox-, IR-), a high proportion of TE-associated genes (71%, 56%, 77%) fell into significantly enriched biological processes, predominantly related to cell cycle/mitosis, repair, mitochondrial function, energy metabolism, and RNA processing (Fig. 5f; Supplementary Fig. 5g). The most recurrently associated genes (e.g., ORC3, SUCLA2, PHB2) aligned with these categories (Supplementary Fig. 5h), suggesting that control groups retained a more coherent transcriptomic state. Under senescence conditions ( > P8, Dox + , IR + ), this proportion dropped markedly to 12%, 14%, and 11%, respectively, with no coherent GO term enrichment or hub-gene patterns emerged (Supplementary Fig. 5e). Consistent with increased transcriptional heterogeneity in senescence, dispersion analyses showed significant increases in senescence-associated transcriptional heterogeneity in late-passage, Dox + , and IR+ groups (Supplementary Fig. 5f).

### Key TEs recurrently associated with transcriptomic signatures related to hADSC states

**Fig. 6:**
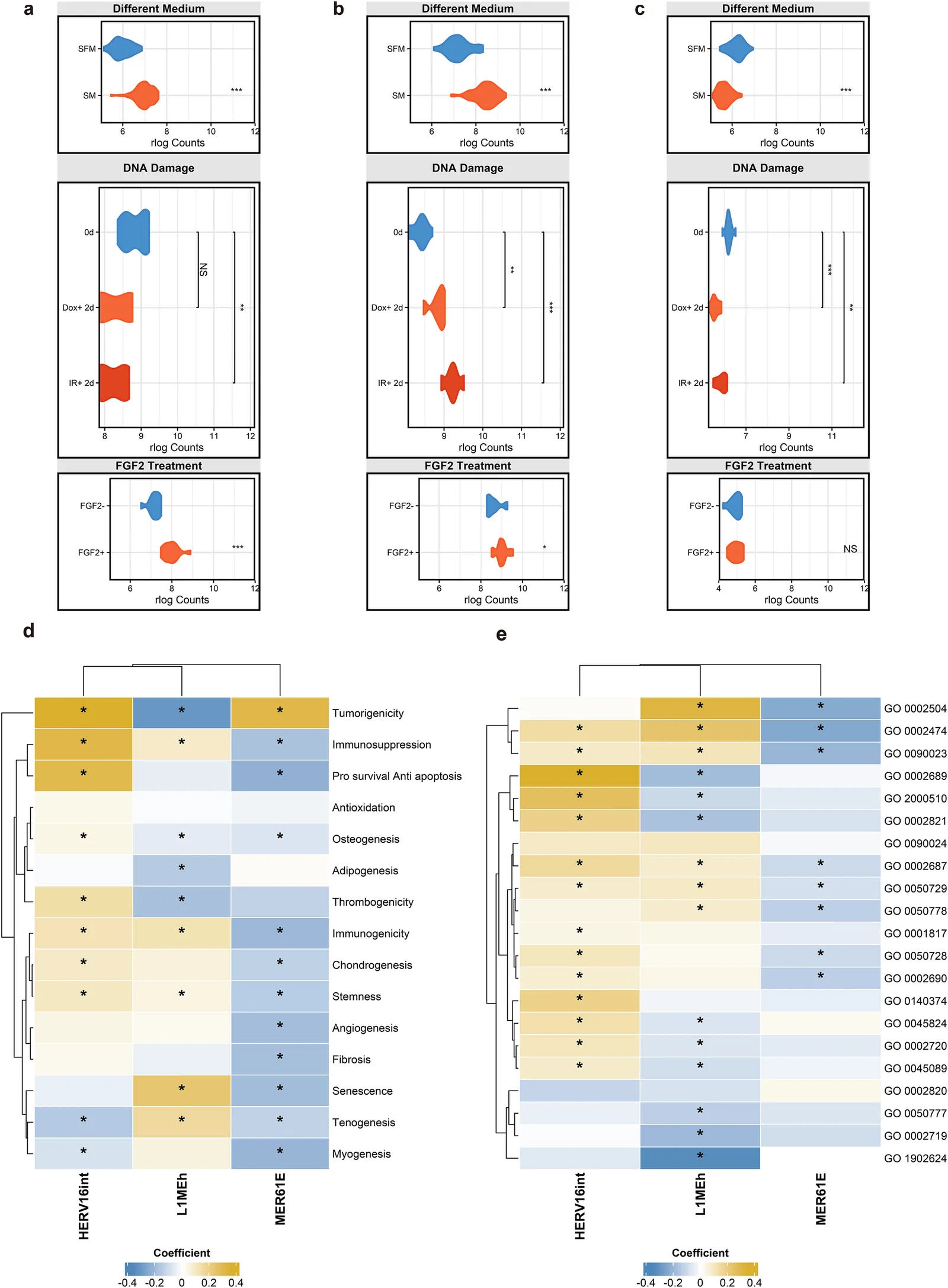
Associations of three highlighted TEs with hADSC transcriptional signatures. Horizontal violin plots showing expression of HERV16-int (**a**), L1MEh (**b**), and MER61E (**c**) across culture medium, DNA-damage, and FGF2 conditions. Expression is shown as regularized log-transformed counts (rlog counts). In the medium panels, serum-free medium (SFM) and serum-containing medium (SM) are shown in blue and orange, respectively; in the DNA-damage panels, 0 h, Dox 2 d, and IR 2 d are shown in blue, orange, and red, respectively; in the FGF2 panels, FGF2− and FGF2+ are shown in blue and orange, respectively. Statistical significance is indicated in the plots (***P ≤ 0.001, **P ≤ 0.01, *P ≤ 0.05, NS, not significant). **d** Heatmap showing associations between the three highlighted TEs and mesenchymal stem cell (MSC)-related transcriptional signatures. **e** Heatmap showing associations between the three highlighted TEs and immunobiology-related gene sets. In **d** and **e**, colors indicate association coefficients between TE expression and GSVA scores, with yellow and blue indicating positive and negative associations, respectively. Asterisks indicate robust associations supported by cross-validation consistency > 0.8 and 95% bootstrap confidence intervals not crossing zero.

Across analyses, three TEs were repeatedly identified as environmentally responsive DETEs with distinct patterns across experimental settings: HERV16-int, L1MEh, and MER61E. HERV16-int expression was significantly upregulated in SM and FGF2 + , but downregulated in Dox+ and IR+ groups (Fig. 6a); L1MEh was significantly upregulated in SM, FGF2 + , Dox + , and IR+ groups (Fig. 6b). MER61E was significantly upregulated in SFM but downregulated in Dox+ and IR+ groups (Fig. 6c).

To examine transcriptomic programs associated with these TEs, we used a curated collection of gene sets representing MSC-related transcriptional features (Supplementary Table 6). Within the analyzed cohort, TE expression levels were associated with GSVA scores of these predefined feature-related gene sets. We evaluated the robustness of these TE-signature associations using cross-validation (CV) and bootstrap resampling (Supplementary Table 7; Fig. 6d). HERV16-int expression was positively associated with GSVA scores for gene sets annotated as tumorigenicity-related, immunosuppression-related, pro-survival/anti-apoptosis-related, osteogenesis-related, thrombogenicity-related, immunogenicity-related, stemness-related, and chondrogenesis-related, and negatively associated with tenogenesis-related and myogenesis-related gene sets. L1MEh expression was positively associated with immunosuppression-related, immunogenicity-related, stemness-related, senescence-related, and tenogenesis-related gene sets, but negatively associated with tumorigenicity-related, osteogenesis-related, and adipogenesis-related gene sets. MER61E expression was positively associated with tumorigenicity-related gene sets, but negatively associated with immunosuppression-related, pro-survival/anti-apoptosis-related, osteogenesis-related, immunogenicity-related, chondrogenesis-related, stemness-related, angiogenesis-related, fibrosis-related, senescence-related, tenogenesis-related, and myogenesis-related gene sets. These results refer to annotated transcriptional signatures rather than directly measured cellular phenotypes or functional endpoints.

Given the prominence of immunomodulatory programs in MSC biology, we further assessed immune-related processes using the same approach (Supplementary Table 8; Fig. 6e). HERV16-int expression was positively correlated with GSVA scores for inflammatory response regulation (GO:0050728, GO:0050729), positive regulation of adaptive immune response (GO:0002821), innate immune response regulation (GO:0140374, GO:0045824, GO:0045089), regulation of cytokine (GO:0001817, GO:0002720), immune cell regulation (GO:0002687, GO:0002689, GO:0002690, GO:0090023, GO:2000510) and antigen processing and presentation of peptide antigen via MHC class I (GO:0002474). Notably, HERV16-int was also positively associated with GSVA scores for viral immune response processes (GO:0140374), which is consistent with the annotation of HERV16-int as an endogenous retroviral element. L1MEh was positively correlated with GSVA scores for MHC I and MHC II antigen processing and presentation (GO:0002474, GO:0002504), positive regulation of inflammatory response and regulation of global immune response (GO:0050729, GO:0050778), leukocyte migration and neutrophil chemotaxis (GO:0090023, GO:0002687), but negatively correlated with GSVA scores for negative regulation of adaptive immune response and positive regulation of neutrophil migration. In contrast, MER61E showed predominantly negative associations across the immune signatures examined.

## Discussion

Early studies reported that among commonly studied ASCs, only neural stem cells exhibit high LINE1 transcriptional activity [31]. Nonetheless, accumulating evidence suggests that TEs may influence ASC biology by modulating key cellular properties [31, 42, 43]. These observations underscore the need to more systematically evaluate TE activity during ASC biomanufacturing and to determine its potential implications for therapeutic efficacy and safety, pending functional validation. In the present study, we provide an integrated view of TE expression patterns in hADSCs across multiple biologically and manufacturing-relevant contexts, based on our in-house multi-module cohort together with complementary external/public hADSC datasets generated from independent donor backgrounds. Similar to protein-coding genes, the TE transcriptome exhibited computational discriminative capacity in distinguishing cellular states of hADSCs. Overall, TE expression was highly plastic and responsive to environmental cues, likely reflecting differences in epigenetic and metabolic states influenced by culture media composition, growth factor exposure, and senescence-associated conditions. Together, these findings potentially raise an underexplored regulatory dimension in ASCs that warrants further investigation. In principle, TE dynamics could serve as transcriptional indicators relevant to ASC identification and function- and safety-related considerations during biomanufacturing and applications.

To obtain MSCs with desired biological characteristics and safety features, substantial efforts have been devoted to defining optimal media composition for in vitro MSC culture [40, 44]. Traditional culture systems often include serum; however, serum use raises well-recognized concerns [40]. In this study, SM was associated with upregulation of several TEs, including HERV16-int and L1MEh, which were linked to transcriptional programs related to immunogenic and immunosuppressive features. In contrast, MER61E was upregulated under SFM conditions and was negatively associated with GSVA scores for multiple MSC-related transcriptional signatures with the exception of tumorigenicity-related transcriptional programs. In addition, SFM-associated TE expression patterns were linked to transcriptional signatures related to proliferation and telomere maintenance. Collectively, these results suggest that culture medium composition is associated with distinct TE expression patterns and corresponding transcriptional states, which may be relevant to transcriptional states involved in MSC maintenance.

Growth factors are essential components of ASC culture and can influence cellular states and properties. FGF2 is widely used to support MSC proliferation and stemness [45]. We identified 90 TEs associated with FGF2 exposure that showed consistent distributions across experimental settings. Notably, FGF2-associated TE-expression patterns were repeatedly observed across the analyzed settings, including Dox or irradiation conditions. These data suggest that FGF2-associated TE programs may reflect transcriptional states linked to cellular responses under defined culture conditions, and raise the possibility that specific TEs could serve as indicators of growth factor-associated transcriptional states.

Donor heterogeneity remains a central challenge but rarely explored for MSC translation. We observed donor-associated variability in ASC TE expression patterns, which may be relevant to future efficacy- and safety-oriented evaluations pending functional validation and warrants further investigation in larger cohorts. Notably, donor-associated TE patterns were modulated by culture conditions, suggesting that donor individuality is plastic and potentially influenced by epigenetic regulation. Consistent with this interpretation, we observed that donor-associated TEs were correlated with pathways related to cellular metabolism (e.g., ribosomal activity, mitochondrial function, and RNA processing), suggesting that variability may reflect differences in donor metabolic environments or states rather than fixed genetic differences alone, a possibility requiring further investigation. These observations suggest that donor-associated variabilities in ASC TE expression could be mitigated through optimization of culture conditions; nonetheless, validation in larger and more diverse donor cohorts is required.

TE expression is frequently dysregulated in senescent cells and has been associated with chromosomal abnormalities and epigenetic alterations [16]. Consistent with prior work, genes associated with TEs activated in senescent conditions (late passage or Dox/IR) did not show coherent functional enrichment in our analyses, consistent with increased variability of TE expression. However, in the DNA damage models, TE shifts likely reflect broad stress-driven transcriptome remodeling rather than a TE-specific response, and functional activation of TEs will require dedicated assays. By contrast, genes associated with TEs expressed in non-senescent hADSCs showed enrichment for biologically interpretable pathways. Further work is needed to determine whether and how TE activation is associated with ASC senescence-related transcriptional changes, and whether variability in TE expression could inform future approaches to ASC quality control.

Finally, we identified three dynamically regulated TEs, HERV16-int, L1MEh, and MER61E, whose expression patterns were associated with hADSC immunomodulation properties, stemness, and/or senescence at the transcriptional level. MER61E has been reported to be expressed in a variety of tumor cell lines, where its expression was correlated with p53 status [46], and in trophoblast cells that have been suggested to exhibit stem cell-like features [47]. It is therefore of interest to experimentally explore whether and how these TEs may be involved in hADSC biology and how their expression patterns relate to future clinical considerations. The unbiased systematic analysis approach employed here provides a tentative framework for prioritizing candidate TEs associated with biological properties of interest in basic research and, potentially, in clinical contexts.

Our analyses relied on transcriptomic profiling and integrative bioinformatics and did not include direct functional assays; thus, the TE–trait relationships reported here are associative and require mechanistic and functional validation. Given the non-orthogonal design, limited donor number, and uneven donor representation across conditions, these results should be viewed as cohort-specific and require confirmation in larger cohorts. Nevertheless, the recurrence of key medium-, FGF2-, and stress-associated TE patterns across internal conditions and external/public datasets argues against donor composition as a major source for the principal findings. In summary, the plasticity of ASC TE expression supports further investigation, and defining TE-associated transcriptional states may help optimize manufacturing and quality-control processes, for example providing candidate TE-expression marker panels for identity, efficacy, or safety-related considerations, as regenerative medicine applications evolve.

## Materials and methods

### hADSC culture and treatment

In this study, all hADSCs used were from our previous studies [48, 49]. After hADSCs were resuscitated, they were directly cultured in the desired medium and incubated at 37 °C with 5.0% CO_2_. For passaging, when cells reached 90% confluency, 0.05% Trypsin/EDTA (Gibco, USA) was used and the resuspended cells were replated at 2–4 × 10^4^ per well.

To test different manufacturing media, hADSCs were plated in DMEM/F12-10% FBS (Gibco, USA), MSC NutriStem® XF Medium (Biological Industries Israel Beit Haemek Ltd, Israel), MesenCult™-ACF Plus Medium (STEMCELL, Canada), and Takara Cellartis® Xeno-Free Basal Medium (Takara Bio Inc., Japan) at a density of 1 × 10^4^ per well in aliquots of each medium. hADSCs were harvested as samples at passages 4, 6, 8, 10, and 12, respectively. Alternatively, hADSCs were seeded in equal numbers at a density of 1 × 10^4^ cells per well and cultured in four media: DMEM/F12 supplemented with 10% FBS, Optvitro MSC Expansion Basal Medium supplemented with Optvitro MSC Proliferation Serum-Free Additive Fraction (ExCell Bio, China), Takara Cellartis® Xeno-Free Basal Medium, and MSC Serum-Free Medium (Yocon, China). All media were supplemented with the same dose of bFGF (FGF2; 40 ng/mL; H&Z Life Science Co., Ltd.) and cells were maintained at 37 °C with 5.0% CO2. Samples were collected at passages 3, 5, 8, and 11 for subsequent RNA sequencing.

For doxorubicin treatment, hADSCs were resuscitated and cultured in DMEM/F12-10% FBS containing doxorubicin (0.2 μM, BBI Life Sciences, China) or DMEM/F12-10% FBS containing doxorubicin (0.2 μM) and supplemented with FGF2 and cultured for a maximum of 2 d. For radiation treatment, hADSCs were resuscitated and cultured in DMEM/F12-10% FBS or DMEM/F12-10% FBS supplemented with FGF2 at the indicated concentration. The hADSCs were then irradiated at 2 Gy for 2 d. Samples were collected at 0 d and 2 d for each treatment for subsequent RNA sequencing.

To investigate the effect of FGF2 on TE expression in hADSCs, hADSCs were cultured in DMEM/F12-10% FBS with or without FGF2 and switched to medium either without or with FGF2 at passage 9. For bafilomycin A1 and rapamycin treatments, we changed the medium to DMEM/F12-10% FBS containing the indicated doses of bafilomycin A1 or rapamycin at passage 9. Cells at passage 9, 10, and 11 were harvested for subsequent RNA sequencing.

A total of 95 hADSC samples were analyzed in this study. Samples were generated under multiple experimental and biological conditions, including culture medium formulation (serum-containing medium, SM, *n* = 68; serum-free medium, SFM, *n* = 27), FGF2 supplementation status (FGF2 + , *n* = 46; FGF2-, *n* = 49), donor identity (four independent donors: D1, *n* = 40; D2, *n* = 20; D3, *n* = 24; D4, *n* = 11), passage-dependent replicative aging ( ≤ P8, *n* = 48; >P8, *n* = 47), and senescence-inducing DNA damage (doxorubicin and/or irradiation, *n* = 12) as shown in Supplementary Table 3.

### Study design and potential donor confounding

Because samples were assembled from multiple experimental modules and donors, the study design was not fully factorial and donor representation was uneven across conditions; therefore, medium- and FGF2-associated effects should be interpreted as cohort-level associations under the tested settings rather than fully independent factor effects, and donor composition is a potential confounder in factor-level comparisons. To mitigate donor confounding, we used three complementary strategies: (i) cross-module replication for medium effects (Med1/Med2 and external/public datasets), (ii) direction-consistency checks across FGF2 add/remove and perturbation settings, and (iii) donor-stratified analyses within a baseline subset (serum-containing medium without strong perturbation). Residual donor confounding cannot be fully excluded.

### RNA extraction and sequencing

In brief, total RNA was isolated using TRIzol reagent (Invitrogen, USA). RNA integrity was determined using an Agilent 2100 Bioanalyzer (Agilent; Palo Alto, CA, United States). RNA quantity was determined using a NanoDrop (Thermo Fisher Scientific, Wilmington, DE). An aliquot of 2 μg RNA per sample was used for RNA-seq library construction. A sequencing library was created for the RNA library using the NEBNext® Ultra RNA library for Illumina (Biolab, England), according to the manufacturer’s recommendations. The Qubit® RNA Assay Kit (Thermo Fisher Scientific) was used for the initial quantification and diluted to every microgram/microliter. The insert size was evaluated using an Agilent Bioanalyzer 2100 and accurately quantified using the StepOnePlus™ Real-Time PCR System (Thermo Fisher Scientific) with an effective library concentration >10 nM. Index-encoded sampling was performed using the HiSeq PE Cluster Kitv4-cBot-HS (Illumina) in the cBot cluster generation system as described by the manufacturer. After clustering was completed, sequence analysis was performed using Illumina Hiseq 4000, resulting in 150 bp paired-end data.

### TE read detection and counting

TE expression was analyzed using standard procedures recommended by TEtranscripts software (v2.2.1) [50]. Briefly, fastq files of all collected samples were quality controlled, and adapter-trimmed and quality-filtered using trim-galore (v0.6.10). Reads were mapped to the hg38 Homo sapiens genome using the STAR aligner (v2.7.10a) [51]. Samples with a total alignment rate exceeding 85% were selected for analysis. TE reads and gene reads were further counted using the TEtranscripts count mode. TE lengths and copy numbers are provided in Supplementary Table 1. TE expression estimates represent subfamily-level aggregated signals and do not resolve locus-specific transcription or activity.

### Transcriptome data analysis and data statistics

All custom statistical analyses and visualization of results were performed using the packages in R 4.2.2. The DESeq2 R package (v1.42.1) was used for normalization of detected genes and TE counts and differential expression analysis. Data integration was performed using sva (v3.35.2) ComBat-seq function. PCA analysis was done using stats (v4.2.2) prcomp function, and PERMANOVA was performed using vegan (v2.6-4). The umap (v0.2.10.0) was used for UMAP. Intersection analysis as well as visualization was done using venn (v1.11), VennDiagram (v1.7.3), ggvenn (v1.1.10), and ggVennDiagram (v1.2.2). GSVA scoring was performed using the GSVA package (v1.46.0), and variance decomposition analysis was performed using the variancePartition package (v1.28.9). All visualizations were performed with ggplot2 (v3.5.0) as well as derivative packages and pheatmap (v1.0.12). A BH-adjusted *p* value < 0.05 was considered significant. Statistical tests were selected based on the underlying data structure and distribution, and non-parametric or distribution-free methods were used where appropriate.

### Identification of stably expressed transposable elements

To identify the most stably expressed TEs, we utilized a residual-based variability analysis on rlog-normalized data (filtered for mean expression > 1) to decouple expression stability from the technical mean-variance dependency. We modeled the expected noise trend by fitting a non-parametric local regression (LOESS, span = 0.75) to the log-transformed row means (μ) and variances (${\sigma }^{2}$) according to the equation ${\log }_{10}({\sigma }^{2}{)}_{{fitted}}={f}_{{loess}}({\log }_{10}(\mu ))$, and subsequently quantified stability using the variability score: ${Residual}={\log }_{10}({\sigma }^{2}{)}_{{observed}}-{\log }_{10}({\sigma }^{2}{)}_{{fitted}}$ TEs were ranked in ascending order based on these scores, where the lowest (most negative) residuals represented the highest expression stability.

### Biological function enrichment analysis of transposable element-associated genes (TEBFE)

To explore association patterns between DETEs and host genes, we performed a global Pearson correlation analysis. For each TE, correlation coefficients (r) and *p* values were calculated against all detectable and filtered genes (mean rlog-normalized count > 1). Multiple testing correction was applied using the Benjamini-Hochberg (BH) method to obtain the false discovery rate (FDR). Significant TE-gene pairs were identified based on the following criteria: (1) FDR < 0.05, and (2) ranking within the top 300 pairs by the absolute value of the correlation coefficient ( | ρ | ). All data processing and statistical analyses were conducted in the R statistical environment (v4.2.2) using the tidyverse (v1.3.2) suite.

To summarize directionally grouped TE–gene associations, we integrated DETE results with correlation directionality. TE–gene pairs were classified into two association patterns: an activated association pattern (upregulated TE with positive correlation or downregulated TE with negative correlation) and a suppressed association pattern (upregulated TE with negative correlation or downregulated TE with positive correlation).

Functional enrichment analysis for Gene Ontology (GO) Biological Processes (BP) was performed for each TE-associated gene set using the clusterProfiler package (v4.6.2) with the filter threshold BH-adjusted *p* value ≤ 0.05 and gene number ≥4. Semantic similarity clustering of the identified GO terms was visualized using the simplifyEnrichment package (v4.6.2) [52] to reduce redundancy. To identify pathways that were significantly and recurrently modulated across the TE landscape, rather than specific to a single TE, we developed a recurrence statistical model.

For each GO term, a one-tailed Binomial test was performed to evaluate if the observed frequency of the pathway appearing across different TEs was significantly higher than random expectation. The theoretical probability (${P}_{{theory}}$) was derived from the background gene ratio (BgRatio) provided by the enrichment results. *P* values from the binomial test were adjusted using the BH method. Pathways with an adjusted *p* value ≤ 0.05 were considered significant recurrent pathways.

Key “Hub” genes within the association network were identified based on their degree centrality—specifically, the frequency of significant correlations with differentially expressed TEs. Genes were ranked by the number of unique TEs they were significantly correlated with (FDR < 0.05, absolute value of the correlation coefficient in top 300). The top-ranked 5 genes were selected as potential core targets. The definitions of activated and suppressed representative recurrently associated genes are the same as mentioned above. A TE-Gene interaction network (TE–gene association network) was constructed for the top representative recurrently associated genes using the igraph (v2.1.1) and tidygraph (v1.2.2) packages. The network topology was visualized using ggraph (v2.1.0), employing a focus layout to highlight the connectivity between the top representative recurrently associated genes and their associated TEs.

### Gene set dispersion statistics

To evaluate the global transcriptional stability of senescence-associated pathways, we curated a comprehensive gene set comprising 160 genes derived from the GO terms “Replicative Aging (GO:0090399)” and “Cellular Senescence (GO:0090398),” supplemented by a literature-curated list of MSC senescence markers (Supplementary Table 6). We quantified transcriptional noise by analyzing the inter-group variance of the gene set using a modified Levene’s test approach.

To eliminate the bias caused by differences in absolute expression levels between genes, the normalized expression matrix (rlog-transformed) of the 160-gene set was scaled row-wise (gene-wise). This transformation sets the mean expression of each gene to 0 and the standard deviation to 1 across all samples, allowing for the direct comparison of expression fluctuations rather than absolute abundance. The Z-score transformed data were pooled by experimental group (e.g., ≤P8 vs. >P8, Control vs. IR + /Dox + ). Levene’s test for equality of variances was employed to assess whether the dispersion of gene expression significantly differed between groups. The test statistic was calculated using the car package (v3.1-3) in R.

We calculated the standard deviation (SD) of the Z-scores for each group. The degree of transcriptional perturbation was quantified as the Variance Ratio, defined as

$${{Var}}_{{treatment}}/{{Var}}_{{control}}$$

calculated as

$${{SD}}_{{treatment}}^{2}/{{SD}}_{{control}}^{2}$$

The distribution of Z-scores was visualized using combined violin and box plots. A statistically significant increase in variance (Levene’s test *P* < 0.05) combined with a Variance Ratio > 1 indicates a loss of transcriptional fidelity and increased system-level disorder in the senescent or damaged state.

### Association analysis between TEs and pathway-level transcriptional signatures

To investigate the statistical associations between specific TE-expression levels (HERV16-int, MER61E, and L1MEh) and biological pathway gene signatures, particularly immunological signatures, we employed a robust statistical modeling approach. Pathway-level scores were calculated using GSVA with the GSVA package, utilizing 15 MSC functional marker gene sets and specific immune-related GO terms retrieved via biomaRt (v2.58.2) and org.Hs.eg.db. Multiple linear regression models were constructed to quantify the contribution of TE expression to pathway enrichment scores. To ensure the robustness and statistical validity of the associations between TE expression and immune pathway signatures, we implemented a rigorous dual-validation strategy combining 5-fold cross-validation (CV) and bootstrap resampling.

First, for model evaluation, the dataset was randomly partitioned into 5 non-overlapping folds using the caret package (v7.0-1). In each iteration, a linear regression model was trained on 4 folds and evaluated on the held-out fold. We calculated the coefficient consistency across folds (defined as the proportion of folds sharing the same coefficient sign) to verify that the direction of regulation was not driven by outliers.

Second, for statistical inference, we applied non-parametric bootstrapping with 1,000 iterations using the boot package (v1.3-31). Significance was determined based on 95% percentile confidence intervals (CIs).

An association was considered robust and significant only if it met two criteria: (1) the 95% bootstrap CI did not cross zero, and (2) the direction of the regression coefficient was consistent across > 80% of the CV folds.

Supplementary information is available at Cell Death Discovery website.

## Supplementary information

Supplementary_Figure

Supplement_table_1

Supplement_table_2

Supplement_table_3

Supplement_table_4

Supplement_table_5

Supplement_table_6

Supplement_table_7

Supplement_table_8

## Data Availability

The public data that support these findings can be found on the Gene Expression Omnibus or European Nucleotide Archive website under the accession number: E-MTAB-9979, GSE86244, GSE116815, GSE141708, GSE159137, GSE192750, GSE199826, GSE226232. The raw RNA-seq data generated in this study have been submitted to the Genome Sequence Archive for Human (GSA-Human) at the National Genomics Data Center (NGDC) under accession number HRA019337.
