# Supplementary material for: The landscape and plasticity of transposable element expression in an adult stem cell of high physiological and clinical significance
Source: Cell Death Discov. 2026 Aug 4;12:434. doi: 10.1038/s41420-026-03277-7 (PMC13720130; doi:10.1038/s41420-026-03277-7)
Supplement: Supplementary file 1 [file 41420_2026_3277_MOESM1_ESM.docx]

**Supplementary Figure 1: Data capture process and basic features.**

(a): Experimental design and data collection schematic. (b): Box plots of overall comparison rates for all datasets. Only samples with a comparison ratio greater than 0.85 are retained. (c): The overall expression of housekeeping genes (HKGs) after integration of all datasets. (d): Box plots of MSC markers gene expression. Dotted line (green) y=0.75*max gene means; (orange) y=0.5*max gene means. (e): Stacked bar plot of TEs families and class.

**Supplementary Figure 2: Medium affects TE expression patterns in hADSCs**

(a): PCA showed significant differences in gene expression in hADSC samples cultured in different media. (b): Heat map of TE overall expression’s euclidean distances between all public data samples. (c): Venn diagram showing common differences obtained between different groups TE. B: BI, D: DF12+FBS, E: Excell, S:StemCell, T: Takara, Y: Yocon. (d): Heatmap shows comparison in the SM and SFM groups, |log2FC| > 0.4 TEs. (e): Umap plot shows the comparison in the SM and SFM groups. (f): enriched GO Biological Process (BP) pathways associated with TE-mediated regulatory patterns. The theoretical probability was derived from the background gene ratio (BgRatio) of the enrichment results. Bars represent the −log10(BH-adjusted p value) of each recurrent GO BP term. Only pathways with adjusted p value ≤ 0.05 are shown. (g): TE–gene interaction network of activated and suppressed hub genes. Hub genes were identified based on degree centrality, defined as the number of unique DETEs significantly correlated with each gene (FDR < 0.05; absolute correlation coefficient ranked within the top 300). The top five genes were selected as hub genes. Nodes represent genes or TEs, and edges indicate significant correlations.

**Supplementary Figure 3: FGF2 affects hADSCs TE expression patterns.**

(a): Heatmap shows the expression of 90 DETEs in the transition treatment group (FGF2 to none, none to FGF2). (b, c): enriched GO BP pathways associated with TE-mediated regulatory patterns and TE–gene interaction network of activated and suppressed hub genes. Top: FGF2+, bottom: FGF2-.

**Supplementary Figure 4: Donor-specific features of TEs.**

(a): Heatmap of up- and down-regulated TEs for specific expression in 4 donors. (b): Bar graph showing the types of TEs expressed specifically by the four donors. (c): PCA characterization of overall TE after removal of specifically expressed TEs. (d): PCA shows TE expression profiles that retain only donor-specific TEs. (e): Plot of GO BP enrichment results for TE-associated genes with donor stable TEs. (f): Plot of GO BP enrichment results of associated genes for 4 donor-specific TEs.

**Supplementary Figure 5: TE expression pattern with different cellular senescence models.**

(a): PCA shows the global features of the gene expression of Dox+/- and IR +/- groups samples. (b): PCA shows ≤P8 and > P8 groups samples global features of TE in Med1. (c): Heatmap showing the expression patterns of 53 differentially expressed transposable elements (DETEs) during replicative senescence. (d): Box-and-violin plots show the distributions of log2 fold changes for differentially expressed protein-coding genes and TE features under Dox, IR, and replicative senescence (RS) conditions (padj < 0.05). (e): Bar plot shows the ratio of TEs-related genes that can be enriched for meaningful functions (Gene counts > 5, adjusted P < 0.05). (f): Gene set dispersion analysis of senescence-associated genes. Violin and box plots show the distribution of Z-score–normalized expression values of a curated senescence-associated gene set (n = 160) across experimental groups. Transcriptional dispersion was quantified by comparing inter-group variances using Levene’s test. Group-wise standard deviations (SD) and the variance fold change (Var_treatment / Var_control) are annotated. A significant increase in variance (Levene’s test P < 0.05) together with a variance ratio > 1 indicates increased transcriptional noise and reduced transcriptional fidelity in the senescent state. (g): enriched GO BP pathways associated with TE-mediated regulatory patterns. (h): TE–gene interaction network of activated and suppressed hub genes.

**Supplementary Figure 6: 3 specific TEs can be associated with MSCs phenotype.**

(a): Umap plot shows the distribution of sample features in all samples. (b): 3 Expression distribution of TE in all samples. (c): Umap plot shows gene set GSVA scoring results for 15 MSCs features.
